# Benefit of prophylactic pelvic irradiation in intermediate-risk prostate cancer: A multicenter retrospective study (iPPAPI)

**DOI:** 10.1016/j.ctro.2025.101093

**Published:** 2025-12-06

**Authors:** Charles Raynaud, Rafik Nebbache, Yazid Belkacemi, Cyrus Chargari, Catherine Durdux, Christophe Hennequin, Florence Huguet, Laurent Quero, Jean-Emmanuel Bibault

**Affiliations:** a Radiation Oncology Department, Hôpital Européen Georges Pompidou, Paris, France; b Radiation Oncology Department, Institut Gustave Roussy, Villejuif, France; c Radiation Oncology Department, Hôpital Mondor, Creteil, France; d Radiation Oncology Department, Hôpital Pitié-Salpêtrière, Paris, France; e Radiation Oncology Department, Hôpital Saint Louis, Paris, France; f Radiation Oncology Department, Hôpital Tenon, Paris, France; g INSERM UMR 1138, Centre de Recherche des Cordeliers, Paris, France

**Keywords:** Radiotherapy, Prostate cancer, Whole Pelvic Radiotherapy, GI toxicity, Intermediate risk

## Abstract

Context.

Prostate cancer (PCa) is the most common urologic malignancy in men, with most cases diagnosed at a localized stage. The benefit of whole-pelvic radiotherapy (WPRT) to eradicate subclinical nodal disease remains debated, particularly in intermediate-risk PCa. This study assessed the impact of WPRT in this population.

**Methods:**

We conducted a multicenter retrospective study within the AP-HP GRRAP program across five radiotherapy departments. Biopsy-proven intermediate-risk PCa (d’Amico classification) treated with conformational external beam radiotherapy between 2010 and 2019 were included. The primary endpoint was recurrence-free survival (RFS), defined as time from diagnosis to biochemical, local, metastatic recurrence, or death. Secondary endpoints were overall survival (OS) and acute (<6 months) or late (≥6 months) genitourinary (GU), gastrointestinal (GI), and sexual toxicities (CTCAE v4.03/v5.0). Survival outcomes were assessed using univariate and multivariate Cox models.

**Results:**

Three hundred patients (60 per center) were included; 94 % received IMRT and 6 % 3D-RT. After a median follow-up of 77 months, univariate analysis showed no significant association between WPRT and RFS (HR; 0.61; 95 % CI, 0.26–1.42; p = 0.25). Multivariable analysis adjusted for clinical and treatment factors yielded similar results (HR, 0.70; 95 % CI, 0.27 to 1.81; *p* = 0.46). OS results were also comparable. Rates of grade 2 acute or late toxicities were similar, but grade ≥ 3 late GI toxicity was higher with WPRT (14.3 % vs. 5.4 %; p = 0.045; OR 2.90).

**Conclusion:**

In intermediate-risk PCa, WPRT did not improve RFS or OS compared with prostate-only RT but was associated with an increased risk of severe GI toxicity.

## Highlights

• WPRT did not show an improvement in relapse-free or overall survival in intermediate-risk patients.
• Acute GU, GI, and sexual toxicities were similar between WPRT and PORT.
• Severe late GI toxicity occurred more frequently with WPRT.
• Findings should be interpreted cautiously due to the retrospective study design.
• Prospective trials and predictive tools are needed to identify patients who may benefit from WPRT.

## Introduction

Prostate cancer (PCa) is the most common urological malignancy in men. In France, data from GLOBOCAN 2022 indicate an incidence of 84.5 per 100,000 men, and mortality of 8.7 per 100,000 men [1], [2]. In 99 % of cases, PCa is an adenocarcinoma, and the median age at diagnosis is 67 years [3].

Approximately 93 % of PCa cases are diagnosed at a localized stage, with 10-year specific survival rates of 95 % [4].

According to the European Society for Medical Oncology (ESMO) guidelines, risk stratification of PCa is performed using the d’Amico classification. In cases of unfavorable intermediate- or high-risk disease, additional staging is recommended using either prostate-specific membrane antigen positron emission tomography (PSMA PET) or a combination of bone scan and thoraco-abdominopelvic computed tomography (CT) [5].

Management of localized PCa includes several evidence-based treatment options. Radiotherapy (RT), possibly combined with androgen deprivation therapy (ADT), is one of the standard curative approaches. In patients with low-risk or favorable intermediate-risk disease, active surveillance is also widely accepted. Radical prostatectomy remains another definitive curative option [5], [6].

One of the most debated issues in the radiotherapeutic management of prostate cancer is whether whole-pelvis radiotherapy (WPRT) can eradicate subclinical nodal metastases and improve cancer-specific outcomes. Although several trials have explored this question and recent data support the use of WPRT in high-risk patients, its role in the intermediate-risk population remains uncertain. The distinction between favorable and unfavorable intermediate-risk disease further complicates decision-making in this setting as these subgroups differ substantially in their baseline risk of nodal involvement and biochemical relapse, which may influence both the absolute benefit expected from WPRT and the risk of added toxicity. To date, no prospective randomized trial has specifically investigated this question, leaving this subgroup as a clinical gray zone where definitive treatment recommendations are lacking. This study aims to evaluate the impact of WPRT in patients with intermediate-risk PCa based on our institutional cohort.

## Material and methods

### Patients

This study was conducted as part of the GRRAP group (Groupe de Recherche en Radiothérapie de l’Assistance Publique − Hôpitaux de Paris), which encompasses all five RT departments within the Assistance Publique-Hôpitaux de Paris (AP-HP) network (Georges Pompidou European Hospital − EGP, La Pitié-Salpêtrière Hospital, Saint-Louis Hospital, Tenon Hospital and Mondor Hospital). We retrospectively included all patients treated with conformal external beam RT (EBRT), either 3D-RT or intensity modulated radiotherapy (IMRT) between January 2010 and December 2019 for biopsy-confirmed localized PCa. Eligibility was limited to patients with intermediate-risk disease, according to the d’Amico classification defined as T2b stage (according to the 7th edition of the TNM classification, in use from 2010 to 2016, and the 8th edition implemented after 2016, with no notable changes in the T-stage definitions between the two versions) or ISUP score of 2 or 3 [GS = 7] [11] or PSA > 10 and < 20 ng/mL). All patients underwent prostate MRI and a staging workup including bone scintigraphy and thoraco-abdominal-pelvic CT, or 18F-FDG PET/CT when deemed necessary by the treating physician.

A total of 60 patients were consecutively selected from each center, resulting in a final cohort of 300 patients. Only patients with complete baseline information; including initial staging (T, N, and M categories), ISUP grade, PSA level, treatment modalities (including use and duration of androgen deprivation therapy), and medical records with at least 18 months of follow-up after treatment; were eligible for inclusion. The study was approved by the Comité d’Éthique de la Recherche de l’AP-HP (Assistance Publique-Hôpitaux de Paris Institutional Review Board), n° IRB: IORG0010044.

### Main outcomes

The primary endpoint was RFS, defined as the time from diagnosis to any type of recurrence: biochemical (defined as a rise in PSA > 2 ng/mL above the post-treatment nadir), local, or metastatic or death. Information on imaging protocols at the time of suspected relapse was not prospectively predefined and therefore was not systematically collected in our dataset. However, during the study period (2010–2020), participating centers generally adhered to prevailing national and international guidelines for the evaluation of suspected prostate cancer recurrence, which typically recommended mpMRI for assessment of local relapse and bone scintigraphy or, more recently, PSMA-PET for detection of metastatic disease.

Secondary endpoints included OS and the occurrence of acute (within 6 months after the start of RT), or late (occurring more than six months after RT) genitourinary (GU), GI, or sexual toxicities, classified according to the Common Terminology Criteria for Adverse Events (CTCAE) version 4.03 [12] before 2017 and version 5.0 thereafter [13], with no meaningful differences between the two versions for the toxicities assessed [14]. Toxicities were collected retrospectively from the patients’ medical records. For each patient, the cumulative worst toxicity observed during follow-up was recorded for analysis. Roach score was calculated according to the following formula: (0.667 * (PSA)) + (10 * ((GS) − 6)).

### Statistical analyses

Baseline patient and treatment characteristics were summarized using descriptive statistics. Continuous variables were reported as medians with interquartile ranges, and categorical variables as frequencies and percentages. Descriptive analyses and univariate hypothesis testing were performed with the Python TableOne package, which provides counts, percentages, medians and interquartile ranges, and applies χ2 or Fisher’s exact tests for categorical variables, and t-tests or Mann–Whitney U tests for continuous variables, as appropriate, with corresponding p-values.

RFS and OS were estimated using the Kaplan–Meier method, with 5-year survival rates reported alongside 95 % confidence intervals (CIs). Differences between treatment groups were assessed using log-rank tests.

Cox proportional hazards regression models were used for univariate analyses of RFS and OS to estimate hazard ratios (HRs) with 95 % CIs. Multivariable models included prespecified covariates of established or potential prognostic value (age, PSA, ISUP grade, T stage, Roach score, ADT use and duration, treatment center, RT technique, and prostate dose), selected a priori to reduce overfitting. Potential collinearity between covariates was assessed qualitatively, and no redundancy requiring exclusion was identified.

The proportional hazards assumption was tested with time-dependent interactions and not violated.

Toxicity outcomes were analyzed using χ2 or Fisher’s exact tests, with odds ratios (OR) and 95 % CIs. Because the number of grade ≥ 3 events was insufficient to meet minimal events-per-variable requirements, no multivariable model was fitted for toxicity to avoid unstable or biased estimates. Subgroup and toxicity analyses were prespecified as exploratory; no adjustment for multiple testing was applied, and results should be interpreted cautiously.

A sensitivity analysis restricted to patients who all received ADT was performed to assess the robustness of the results.

All analyses were performed in Python (version 3.13.2) within a Jupyter Notebook environment. A two-sided p-value < 0.05 was considered statistically significant.

## Results

### Patient characteristics

Between January 2010 and December 2019, 300 patients were included in the study. The median age was 72 years. Among them, 59 patients (20 %) had a T1-stage tumor, and 241 (80 %) had a T2-stage tumor (either T2a or T2b). The median PSA level at diagnosis was 8.4 ng/mL (range: 0.03–20; mean: 9.2 ng/mL). Regarding histology, 52 patients (17 %) had a ISUP score of 1, 169 (56 %) of 2, and 79 (26 %) 3. Most patients had a WHO performance status of 0 or 1 (99 %).

**Fig. 1:**
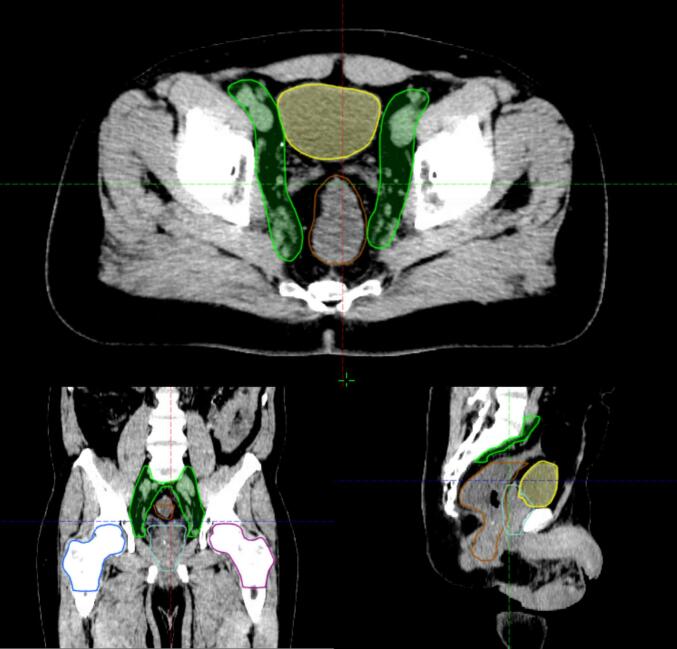
Illustrative example of the pelvic nodal clinical target volume treated.

**Fig. 2:**
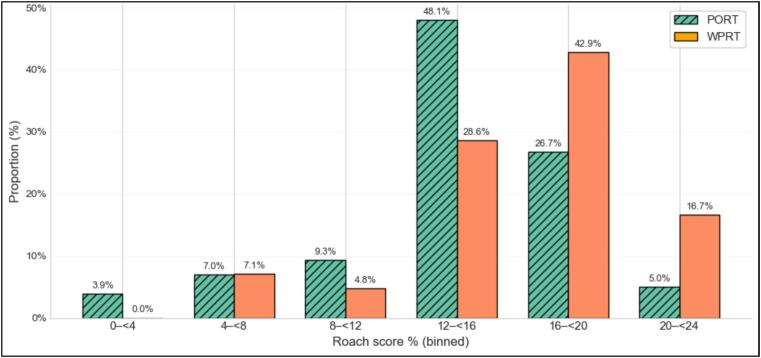
Comparative distribution of Roach scores between the two study cohorts.

Among the included patients, 258 (86 %) received PORT, while 42 (14 %) underwent WPRT (an illustrative example of the pelvic nodal clinical target volume treated is provided in Fig. 1). Compared with the WPRT group, patients treated with PORT had significantly lower median PSA levels at diagnosis (8.0 ng/mL (range: 0.03–20; mean: 8.8 ng/mL) vs. 11.5 ng/mL (range: 3.7–20; mean: 11.3 ng/mL); *p* < 0.05), lower median Roach score (14.7 % vs 17.2 %; *p* < 0.05), and received a significantly lower median radiation dose to the prostate (74 Gy vs. 76 Gy; *p* < 0.05). Patients in the WPRT group were more likely to receive concomitant ADT (*p* < 0.05), and for a significantly longer mean duration (6.3 months vs. 8.9 months; *p* < 0.05). Patients receiving WPRT also had a significantly lower nadir PSA after RT (*p* < 0.05*)*. In the overall cohort, 281 (94 %) of patients received IMRT and 19 (6 %) underwent 3D-RT. These proportions where comparable between the PORT and the WPRT groups with no statistically significant difference observed. A comprehensive summary of baseline patient characteristics is presented in Table 1, and the distribution of Roach scores across the two cohorts is shown in Fig. 2. Differences between centers in terms of patient recruitment and treatment practices are illustrated in Table 2.

**Table 1:** Patient characteristics.

		**Total**	**PORT**	**WPRT**	**p-value**
**n**		300	258 (86 %)	42 (14 %)	
**Age (years), median [Q1,Q3]**		72.2 [68.3,76.4]	72.0 [67.9,76.4]	73.3 [70.3,77.2]	0.157
**WHO performance status, n** **(%)**	0	240 (80.0)	205 (79.5)	35 (83.3)	0.874
	1	57 (19.0)	50 (19.4)	7 (16.7)	
	2	2 (0.7)	2 (0.8)	0 (0.0)	
	3	1 (0.3)	1 (0.4)	0 (0.0)	
**PSA (ng/mL), median [Q1,Q3]**		8.4 [6.3,12.0]	8.0 [6.0,11.3]	11.5 [7.9,13.0]	< 0.001*
**T stage, n (%)**	1	59 (19.7)	54 (20.9)	5 (11.9)	0.248
	2 (a, b)	241 (80.3)	204 (79.1)	37 (88.1)	
**ISUP score, n (%)**	1	52 (17.3)	47 (18.2)	5 (11.9)	0.273
	2	169 (56.3)	147 (57.0)	22 (52.4)	
	3	79 (26.3)	64 (24.8)	15 (35.7)	
**Intermediate risk, n** **(%)**	Favorable	169 (56.3)	147 (57.0)	22 (52.4)	0.697
	Unfavorable	131 (43.7)	111 (43.0)	20 (47.6)	
**Roach score (%), median [Q1,Q3]**		14.9 [13.1,16.8]	14.7 [12.9,16.6]	17.2 [14.2,18.7]	< 0.001*
**Radiotherapy technique**	3D-RT	19 (6.3)	16 (6.2)	3 (7.1)	0.737
	IMRT	281 (93.7)	242 (93.8)	39 (92.9)	
**Radiation dose to the prostate** **(Gy), median [Q1,Q3]**		74.0 [72.5,76.0]	74.0 [72.5,76.0]	76.0 [74.0,76.0]	<0.001*
**Androgen deprivation therapy duration (months), median [Q1,Q3]**		6.0 [6.0,6.0]	6.0 [6.0,6.0]	6.0 [6.0,6.0]	0.003*
**Androgen deprivation therapy, n (%)**	No	108 (36.0)	106 (41.1)	2 (4.8)	< 0.001*
	Short ADT (≤ 6 months)	173 (57.7)	144 (55.8)	29 (69)	
	Long ADT (> 6 months)	19 (6.3)	8 (3.1)	11 (26.2)	
**Nadir PSA (ng/mL), median [Q1,Q3]**		0.1 [0.1,0.4]	0.1 [0.1,0.4]	0.1 [0.0,0.2]	0.037*
**Median follow-up (months)**		77.0 [65.9,94.3]	75.3 [65.4,94.6]	83.0 [72.4,94.1]	0.24

**Table 2:** Baseline patient characteristics and treatment modalities by participating center.

**Center**		**Total**	**Center 1**	**Center 2**	**Center 3**	**Center 4**	**Center 5**
**n**		300	60	60	60	60	60
**Age (years), median [Q1,Q3]**		72.2 [68.3,76.4]	72.1 [68.7,76.2]	74.7 [69.9,76.8]	72.6 [70.1,77.0]	70.7 [66.6,76.2]	71.6 [64.2,75.1]
**PSA, median [Q1,Q3]**		8.4 [6.3,12.0]	8.1 [6.2,10.2]	8.2 [6.5,11.1]	9.9 [6.7,12.3]	9.2 [6.8,13.0]	8.0 [5.7,11.0]
**T, n (%)**	**1**	59 (19.7)	7 (11.7)	6 (10.0)	10 (16.7)	27 (45.0)	9 (15.0)
	**2**	241 (80.3)	53 (88.3)	54 (90.0)	50 (83.3)	33 (55.0)	51 (85.0)
**ISUP, n (%)**	**1**	52 (17.3)	18 (30.0)	6 (10.0)	16 (26.7)	4 (6.7)	8 (13.3)
	**2**	169 (56.3)	33 (55.0)	32 (53.3)	33 (55.0)	40 (66.7)	31 (51.7)
	**3**	79 (26.3)	9 (15.0)	22 (36.7)	11 (18.3)	16 (26.7)	21 (35.0)
**Radiation dose to the prostate, median [Q1,Q3]**		74.0 [72.5,76.0]	76.0 [76.0,76.0]	76.0 [74.0,76.0]	70.0 [70.0,76.0]	72.5 [72.5,72.5]	76.0 [74.0,76.0]
**PORT/WPRT**	**PORT**	258 (86.0)	45 (75.0)	59 (98.3)	46 (76.7)	60 (100.0)	48 (80.0)
	**WPRT**	42 (14.0)	15 (25.0)	1 (1.7)	14 (23.3)	0 (0.0)	12 (20.0)
**Androgen deprivation therapy, n (%)**	**No**	108 (36.0)	34 (56.7)	11 (18.3)	25 (41.7)	33 (55.0)	5 (8.3)
	**Yes**	192 (64.0)	26 (43.3)	49 (81.7)	35 (58.3)	27 (45.0)	55 (91.7)
**Androgen deprivation therapy duration (months), median [Q1,Q3]**		6.0 [6.0,6.0]	6.0 [3.0,6.0]	6.0 [6.0,6.0]	6.0 [6.0,6.0]	6.0 [6.0,6.0]	6.0 [6.0,6.0]

When pelvic nodal regions were irradiated, the median RT dose was 46 Gy. The treated areas included the common iliac nodes (20 %), the external and internal iliac as well as the ilio-obturator regions (100 %), and the presacral region (29 %).

### Outcomes

The median follow-up duration was 77 months. In univariate analysis, WPRT was not significantly associated with RFS (hazard ratio [HR], 0.61; 95 % CI, 0.26 to 1.42; *p* = 0.25). 5 years RFS-rates were 86.5 % [81.6; 90.2] (n = 194) in the PORT group and 95.0 % [81.4; 98.7] (n = 36) in the WPRT group.

**Fig. 3:**
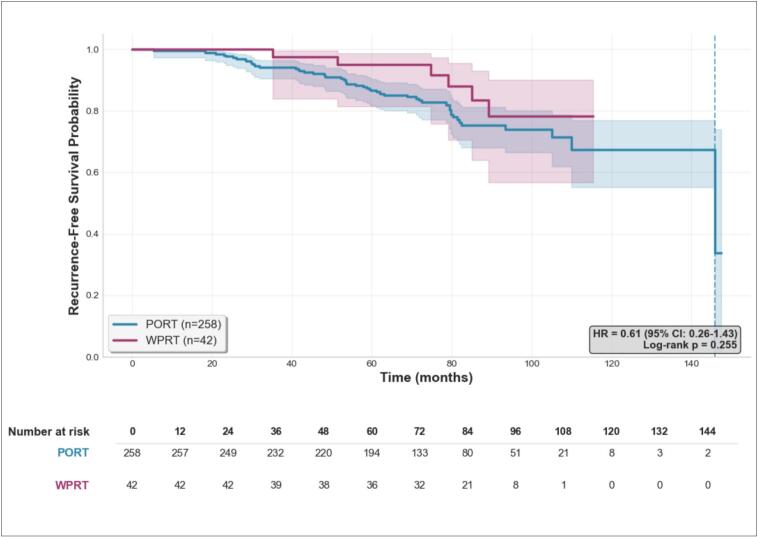
Kaplan–Meier estimates of recurrence-free survival (RFS). Kaplan–Meier curves showing recurrence-free survival according to treatment group (WPRT vs PORT). Shaded areas represent 95% confidence intervals.

After adjustment for prognostic factors: age, baseline PSA, ISUP, T stage, Roach score, ADT (absence, ≤6 months or > 6 months), treatment center, radiotherapy technique and radiation dose to the prostate; multivariable analysis yielded a similar result for RFS (HR, 0.70; 95 % CI, 0.27 to 1.81; *p* = 0.46), confirming the absence of a statistically significant association. Kaplan-Meier estimates of RFS by treatment group are shown in Fig. 3.

**Fig. 4:**
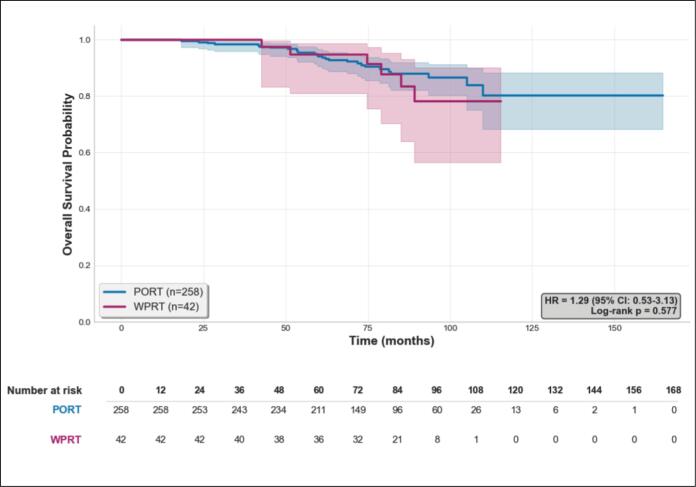
Kaplan–Meier estimates of overall survival (OS). Kaplan–Meier curves showing overall survival according to treatment group (WPRT vs PORT). Shaded areas represent 95% confidence intervals.

WPRT was not found to be significantly associated with OS in univariate analysis (HR, 1.29; 95 % CI, 0.53 to 3.13; *p* = 0.58) nor in multivariate analysis (HR, 1.88; 95 % CI, 0.65 to 5.44; *p* = 0.53). Survival curves for OS are provided in Fig. 4. 5 years OS rates were 94.2 [90.4; 96.5] (n = 211) in the PORT group and 94.9 [81.0; 98.7] (n = 36) in the WPRT group.

The proportional hazards assumption was not violated: no significant time-dependent interaction was observed for RFS (*p* = 0.885) or OS (*p* = 0.552). No obvious redundancy between covariates was observed in the qualitative assessment of collinearity.

A sensitivity analysis was performed by restricting the cohort to patients who all received ADT, given the major impact of this treatment on survival. Results were consistent with the primary analysis, with no significant association between WPRT and RFS (HR 0.61; 95 % CI 0.25–1.52; *p* = 0.30) or OS (HR 1.33; 95 % CI 0.53–3.33; *p* = 0.54).

### Toxicities

The most frequently observed adverse events were GU toxicities, with 42 % of patients experiencing acute grade 2 toxicities and 25 % experiencing grade 2 late toxicities. GI grade 2 toxicities were reported in 13 % of patients during the acute phase and in 8 % during the chronic phase. Regarding sexual toxicities, 6 % of patients reported acute events of grade 2 and 10 % reported late grade 2 events.

Severe toxicities (grade ≥ 3) were generally rare across all domains (<10 %), except for late GI toxicities, which were significantly more frequent in the WPRT group (14.3 % vs. 5.4 %; *p* = 0.045; OR = 2.90). No grade 5 toxicities were reported. A detailed overview of toxicity outcomes is provided in Table 3.

**Table 3:** Toxicity details. Asterisks denote statistically significant differences according to Fisher’s exact test (p < 0.05), GU = genitourinary; GI = gastrointestinal.

**Location**	**Type**	**Total (%)**	**PORT (%)**	**WPRT (%)**	**p-value**	**OR**
GU	Acute = 2	41.7 %	42.6 %	35.7 %	0.50	0.75
	Acute ≥ 3	2.3 %	2.7 %	0.0 %	0.599	∞
	Late = 2	25.0 %	25.2 %	23.8 %	1.000	0.93
	Late ≥ 3	6.3 %	6.6 %	4.8 %	1.000	0.71

GI	Acute = 2	12.7 %	11.2 %	21.4 %	0.079	2.15
	Acute ≥ 3	1.3 %	1.6 %	0.0 %	1.000	∞
	Late = 2	8.3 %	8.1 %	9.5 %	0.763	1.19
	Late ≥ 3	6.7 %	5.4 %	14.3 %	0.045*	2.90

Sexual	Acute = 2	5.7 %	5.8 %	4.8 %	1.000	0.81
	Acute ≥ 3	2.3 %	2.3 %	2.4 %	1.000	1.02
	Late = 2	10.0 %	10.1 %	9.5 %	1.000	0.94
	Late ≥ 3	3 %	3.1 %	2.4 %	1.000	0.76

### Patterns of recurrence

Regarding the sites of recurrence, most patients experienced biochemical relapse 93 % (n = 27), while 45 % (n = 13) had pelvic relapse, 24 % (n = 7) developed metastatic recurrence, and 41 % (n = 12) experienced local failure. Details of the events for both cohorts are provided in Table 4.

**Table 4:** Detail of recurrence and outcome events by cohort.

**Group**	**n**	**Biochemical relapse**	**Pelvic relapse**	**Metastatic relapse**	**Local relapse**	**Deaths**
PORT	258	26 (10 %)	13 (5 %)	6 (2 %)	12 (5 %)	27 (11 %)
WPRT	42	1 (2 %)	0 (0 %)	1 (2 %)	0 (0 %)	6 (14 %)

### Subgroup analyses

For the subgroup analyses, the cohort was stratified into two groups: favorable and unfavorable intermediate-risk disease. Across the two subgroups, no statistically significant differences were observed in univariate analyses for either RFS or OS. Detailed results of these analyses are presented in Table 5.

**Table 5:** Subgroup analyses.

**Subgroup (PORT/WRT)**	**Outcome**	**Events (PORT/WPRT)**	**HR (95 % CI)**	**p-value**
Favorable Risk (147/22)	OS	14/2	0.81 (0.18–3.55)	0.775
	RFS	28/2	0.38 (0.09–1.62)	0.193
Unfavorable Risk (111/20)	OS	13/4	1.81 (0.58–5.63)	0.303
	RFS	25/4	0.84 (0.29–2.43)	0.751

## Discussion

This retrospective study evaluated the long-term outcomes of WPRT in patients with intermediate-risk PCa, as defined by the d’Amico classification. After a median follow-up of 77 months, No significant differences in RFS or OS were observed between the WPRT and PORT groups. Although 5-year PFS numerically favored WPRT over PORT, this difference did not reach statistical significance and should be interpreted cautiously given the limited number of events. For relapse patterns, also acknowledging the small sample size, most failures in both arms occurred as biochemical recurrence, and no pelvic nodal or local failures were observed in the PORT group.

We performed a sensitivity analysis restricted to patients receiving ADT, which suggested that the observed absence of a statistical difference was unlikely to be solely explained by variations in ADT duration between the two treatment groups, although this interpretation should be considered exploratory given the acknowledged limitations of the study. Results were also consistent across both favorable and unfavorable intermediate-risk subgroups.

Regarding toxicity outcomes, our study showed comparable rates of both acute and late GU toxicities between the two arms, and acute GI toxicities were also similar. A slight increase in acute grade 2 GI toxicity was observed in the WPRT arm, but not statistically significant. However, late grade ≥ 3 GI toxicities were significantly more frequent in the WPRT arm, a finding that may partly reflect the higher median prostate dose delivered in this cohort. Moreover, despite different ADT patterns between groups, no difference in late sexual toxicity was observed. This may reflect underreporting of this type of adverse event and/or insufficient statistical power to detect small differences. It should be noted that subgroup and toxicity analyses were based on a limited number of severe events, with wide confidence intervals. No adjustment for multiple testing was applied, and these analyses were predefined as exploratory. Accordingly, their results should be interpreted as hypothesis-generating rather than confirmatory.

Several studies and clinical trials have explored the role of prophylactic pelvic nodal irradiation in PCa. One of the earliest, published in the early 1990 s by Mack Roach et al. [8], proposed a formula derived from the nomogram published by Partin et al. [15] to estimate the risk of nodal involvement. Based on data from 212 patients, this calculation identified two subgroups with low (≈6%) and high (≈40 %) risk of nodal metastases, with a cutoff set at 15 %. Building on this work, the phase III RTOG 9413 trial [9] stratified patient selection according to a Roach score > 15 %. This trial compared four treatment sequences combining or omitting WPRT and ADT, administered either as neoadjuvant-concurrent, or adjuvant treatment. The results indicated that the combination of WPRT with neoadjuvant-concurrent ADT improved RFS compared with PORT combined with neoadjuvant ADT or with WPRT and adjuvant ADT. However, outcomes were similar to those observed with PORT plus adjuvant ADT, thereby introducing uncertainty and leaving the interpretation open to debate [16].

The GETUG-01 trial, conducted by Pommier et al. [17], stratified patients into “low-risk” (T1–T2, Gleason 6, and PSA < 3 × the upper limit of normal) versus “high-risk” (T3 disease, Gleason > 6, or PSA > 3 × upper limit) groups. At five years of follow-up, no significant difference in RFS was observed between WPRT and PORT across risk strata. Long-term follow-up suggested a non-significant trend toward improved RFS in the low-risk subgroup treated with WPRT [7], yet this unexpected trend in the low-risk subgroup makes the overall interpretation of the results more challenging.

More recently, the POP-RT trial, published in 2021 by Murthy et al. [10], focused on high-risk patients defined by a Roach score ≥ 20 %. This study demonstrated a benefit of WPRT over PORT in terms of BRFS and RFS. Importantly, all patients underwent contemporary staging with PSMA-PET, thereby reducing the likelihood of occult metastatic disease at inclusion. Nonetheless, the single-center, single-investigator design may restrict the generalizability of these findings.

Overall, these trials underscore substantial variability in the definition of risk groups across studies. Although some relied on the Roach score, the thresholds used were not uniform. Furthermore, while historically valuable, the Roach formula may overestimate the risk of nodal involvement in patients with T1c–T2 disease in the PSA-screening era [18]. This heterogeneity complicates cross-trial comparisons and underscores the ongoing uncertainty regarding the role of prophylactic pelvic nodal irradiation in intermediate-risk PCa. A detailed overview of these trials is available in Table 6.

**Table 6:** Key randomized trials evaluating prophylactic pelvic nodal irradiation in prostate cancer.

**Trial**	**Patients (period)**	**Risk / Characteristics**	**Design**	**RT technique & Fractionation**	**Main Results**	**Key Toxicity**
**NRG** **/RTOG 9413**	1,322 (1995–1999), 319–321/arm	Roach score > 15 % (high risk)	2 × 2 factorial design: WPRT vs PORT combined with neoadjuvant ADT (NHT) vs adjuvant ADT (AHT); ADT duration: 4 months.	2D RT. Prostate 70.2 Gy/39 fx; Pelvic LN 50.4 Gy/28 fx	10-year RFS: NHT + WPRT 28.4 %, NHT + PORT 23.5 %, WPRT + AHT 19.4 %, PORT + AHT 30.2 %	Late ≥ G3: GI 7 % (WPRT + AHT) vs 2 % (PORT + AHT); GU 7 % vs 4 %

**GETUG 01**	446 (1998–2004), 225 pelvis + prostate vs 221 prostate-only	T1b–T3N0M0. High-risk 78 % (T3 and/or GS7 and/or PSA > ULN). Short-term ADT allowed in high-risk	Phase III randomized: Prostate-only vs Pelvis + Prostate	3D-CRT / 4-field. Pelvis 46 Gy/1.8–2 Gy; Prostate 66 Gy then 70 Gy after 2000 (1.8–2 Gy/fx)	5-year PFS: 66.0 % (Pelvis + Prostate) vs 65.3 % (Prostate-only), p = 0.34. No OS or subgroup benefit	Acute ≥ G3 GU: 3.1 % (Pelvis + Prostate) vs 7.5 % (Prostate-only). Late ≥ G3 GU: 10.8 % vs 12.6 %. Late ≥ G3 GI: ∼7–8 % both arms. No QoL difference

**POP-RT**	224 (2011–2017), 110 WPRT vs 114 PORT	Node-negative, high/very high-risk; pelvic nodal risk ≥ 20 % (Roach); ≥2 years ADT	Phase III single-center randomized 1:1: PORT vs WPRT	Image-guided IMRT with simultaneous integrated boost. Prostate 68 Gy/25 fx (∼2.72 Gy/fx); Pelvic nodes 50 Gy/25 fx	5-year BFFS: 95.0 % (WPRT) vs 81.2 % (PORT), HR 0.23. 5-year DFS: 89.5 % vs 77.2 %, HR 0.40. 5-year OS: 92.5 % vs 90.8 %. 5-year DMFS: 95.9 % vs 89.2 %, HR 0.35	Late ≥ G2 GU: 20.0 % (WPRT) vs 8.9 % (PORT), p = 0.02. Late ≥ G3 GU: 1.8 % both arms. Late ≥ G2 GI: 8.2 % vs 4.5 %, ns. No G4 events. Acute toxicity similar

However, current international guidelines, including the French (AFU [19] and SFRO [20]) and European (EAU [21] and ESMO [5]) recommendations; classify patients based on to the d’Amico risk stratification published in 1998 [22], which distinguishes three groups according to PSA level, clinical T stage, and ISUP. While these guidelines generally support the use of pelvic nodal irradiation in high-risk disease, its role in the intermediate-risk setting remains undefined. Conversely, in our current clinical practice, prophylactic pelvic lymph node irradiation is generally considered appropriate for patients with high-risk or unfavorable intermediate-risk prostate cancer, particularly those with a high estimated risk of nodal involvement (e.g. Roach score ≥ 15–20 %) and/or adverse pathological features such as ISUP ≥ 4, or PSA > 20 ng/mL. In such cases, WPRT is typically combined with long-term androgen deprivation therapy.

Although slightly different in detail, toxicity findings of our study are broadly consistent with the recent published literature [23], [24]. Indeed, the two main contemporary trials reporting toxicity with pelvic irradiation: PIVOTAL [25] and POP-RT [10]; both demonstrated similar rates of acute and late GU toxicities between WPRT and PORT, but consistently observed higher rates of GI toxicity in the WPRT arms. It should be noted, however, that in POP-RT the difference in late GI toxicities did not reach statistical significance. All patients were treated with IMRT in both trials. Moreover, our study also reported sexual toxicities with no differences between the two groups. These adverse events, although rarely documented in clinical trials, have a major impact on patients’ quality of life [26], [27], [28], [29], [30] and are likely underreported in routine practice [31], [32].

Our study has several strengths**.** This multicenter study included 300 patients from five institutions, offering a broad and representative overview of contemporary clinical practice. It specifically addressed the underexplored intermediate-risk subgroup, for which evidence on pelvic nodal irradiation remains limited. The long median follow-up (77 months) enabled a robust assessment of oncological outcomes. Our findings also highlight the substantial heterogeneity in practice patterns for intermediate-risk disease, emphasizing the need for stronger evidence and clearer clinical guidelines in this setting. Finally, all patients were treated between January 2010 and December 2019, a period during which modern radiotherapy techniques such as IMRT (94 % of the cohort) and daily image guidance were widely implemented across participating centers.

Several limitations warrant consideration. The retrospective design exposes the study to selection bias and residual confounding. Although propensity score–based methods such as inverse probability of treatment weighting (IPTW) have been used in other series [33], we did not apply this approach because of the small size of the WPRT arm (n = 42) and the limited number of events, which could yield unstable weights and violate the positivity assumption. Instead, we used adjusted multivariable Cox models.

Practice patterns evolved over the study period and varied across centers, reflecting differences in patient selection, clinician preference, and institutional protocols. The extent of pelvic nodal irradiation also differed: some centers used a limited pelvic field (mainly obturator and internal/external iliac nodes) for intermediate-risk or node-negative patients and reserved extended fields (common iliac ± presacral nodes) for higher-risk cases, whereas others systematically applied an extended field irrespective of risk group. Given the retrospective and multicenter nature of the cohort spanning nearly a decade, we were unable to capture all center-level policy changes or individual clinician decisions. We therefore compared baseline characteristics and treatment variables across centers and included “center” as a covariate in our multivariable models to account for inter-center differences.

Additional variability arose from differences in imaging modalities used for nodal and metastatic staging in unfavorable intermediate-risk disease—CT, FDG-PET, or bone scintigraphy during most of the study period versus PSMA-PET more recently—which may have led to underestimation of nodal involvement at baseline. The definition of T stage also evolved with the widespread adoption of prostate MRI compared with earlier reliance solely on clinical staging.

Finally, despite the overall sample of 300 patients, the relatively favorable prognosis and low recurrence rate of intermediate-risk prostate cancer limited the number of events and, consequently, the statistical power of our analyses. This may have reduced our ability to detect modest but potentially clinically meaningful differences between treatment groups. This limitation particularly applies to subgroup and toxicity analyses, which lack statistical power and must be interpreted with caution.

Ongoing trials such as RTOG 0924 [33], PIVOTAL Boost [34] and PEACE-2 [35] are expected to provide further data regarding the role of WPRT in patients with high-risk or unfavorable intermediate-risk PCa. In parallel, the development of novel predictive approaches, including multi-omic profiling [36], [37] assisted by artificial intelligence, may help refine patient selection for WPRT in the future.

## Conclusion

In this retrospective study, WPRT did not show a statistically significant improvement in RFS or OS compared with PORT in patients with intermediate-risk PCa. WPRT was associated with a higher risk of grade ≥ 3 GI toxicity. These findings should be interpreted with caution given the retrospective design, potential selection biases, the small WPRT sample size and limited statistical power, which preclude definitive conclusions regarding the absence of benefit. Further prospective studies are warranted to clarify the potential role of pelvic nodal irradiation in this setting.

## Declaration of competing interest

The authors declare that they have no known competing financial interests or personal relationships that could have appeared to influence the work reported in this paper.
